# Malnutrition and Risk of Procedural Complications in Patients With Atrial Fibrillation Undergoing Catheter Ablation

**DOI:** 10.3389/fcvm.2021.736042

**Published:** 2021-10-25

**Authors:** Daehoon Kim, Jaemin Shim, Yun Gi Kim, Hee Tae Yu, Tae-Hoon Kim, Jae-Sun Uhm, Jong-Il Choi, Boyoung Joung, Moon-Hyoung Lee, Young-Hoon Kim, Hui-Nam Pak

**Affiliations:** ^1^Division of Cardiology, Department of Internal Medicine, Yonsei University Health System, Seoul, South Korea; ^2^Korea University Cardiovascular Center, Seoul, South Korea

**Keywords:** atrial fibrillation, catheter ablation, complication, malnutrition, rhythm outcome

## Abstract

**Background:** Little is known about the prognostic value of nutritional status among patients undergoing atrial fibrillation (AF) catheter ablation (AFCA). We compared the risk of procedure-related complications and long-term rhythm outcomes of AFCA according to nutritional status.

**Methods:** We included 3,239 patients undergoing *de novo* AFCA in 2009-2020. Nutritional status was assessed using the controlling nutritional status (CONUT) score. The association between malnutrition and the risk of AFCA complications or long-term rhythm outcomes was evaluated. We validated the effects of malnutrition using an external cohort of 360 patients undergoing AFCA in 2013-2016.

**Results:** In the study population (26.8% women, median age: 58 years), 1,005 (31.0%) had malnutrition (CONUT scores ≥ 2); 991 (30.6%) had mild (CONUT 2–4) and 14 (0.4%) had moderate-to-severe (CONUT ≥ 5) malnutrition. The overall complication rates after AFCA were 3.3% for normal nutrition, 4.2% for mild malnutrition, and 21.4% for moderate-to-severe malnutrition. Moderate-to-severe malnutrition [odds ratio (OR) 6.456, 95% confidence interval (CI) 1.637-25.463, compared with normal nutrition], older age (OR 1.020 per 1-year increase, 95% CI 1.001-1.039), female sex (OR 1.915, 95% CI 1.302-2.817), and higher systolic blood pressure (OR 1.013 per 1-mmHg increase, 95% CI 1.000-1.026) were independent predictors for the occurrence of complications. In the validation cohort, malnutrition (CONUT ≥ 2) was associated with a 2.87-fold higher risk of AFCA complications (95% CI 1.174-7.033). The association between malnutrition and a higher risk of AFCA complications was consistently observed regardless of body mass index and sex. Malnutrition did not affect rhythm outcomes during the median follow-up of 40 months (clinical recurrence: 37.0% in normal nutrition vs. 36.5% in malnutrition).

**Conclusion:** Malnutrition, which is common in patients undergoing AFCA, was associated with a substantially higher risk for complications after AFCA.

## Introduction

Malnutrition is prevalent in ~34% of hospitalized patients, even in developed countries (1). Malnutrition has been reflected by a lower body mass index (BMI) in previous studies and has been shown to be associated with higher incidences of atrial fibrillation (AF) and arrhythmia progression, as well as poor prognosis among those with AF (2–5). However, a recent study reported that malnutrition is common in obese patients with heart failure, suggesting the BMI *per se* does not fully reflect the nutritional status (6).

Atrial fibrillation increases the risk of cardiovascular mortality and morbidity resulting from strokes and heart failure and impairs quality of life (7, 8). Compared with antiarrhythmic drug (AAD) therapy, AF catheter ablation (AFCA) reduces the number of acute episodes and prolongs the duration of sinus rhythm, thereby improving the quality of life (9). AFCA has been found to be associated with a lower risk of mortality and hospitalizations for heart failure (10), a lower risk of stroke (11), and improved cognitive function (12, 13). Among the various screening tools for malnutrition, controlling the nutritional status (CONUT) score has been studied in AF (14, 15). Malnutrition determined by this score is an independent predictor of a poor prognosis, especially hemorrhagic adverse events and AF recurrence after AFCA (14, 15). However, few studies have evaluated the effect of nutritional status on the outcomes of AFCA, and the relationship between the nutritional status and safety of AFCA remains unclear.

We previously reported on poor long-term rhythm outcomes after AFCA in patients with metabolic syndrome and a higher pericardial fat volume (16, 17). The aim of the present study was to investigate the association between nutritional status and the efficacy and safety of catheter ablation in patients with AF. In this study, we compared the risk of procedure-related complications and long-term rhythm outcomes according to CONUT score.

## Materials and Methods

### Study Population

The study protocol adhered to the Declaration of Helsinki and was approved by the institutional review board of the Yonsei University Health System. Written informed consent was obtained from all patients included in the Yonsei AF Ablation Cohort Database (NCT02138695). Among 3,375 patients who underwent *de novo* AFCA for symptomatic drug-refractory AF, 3,239 patients who had available data on their serum albumin, cholesterol, and total lymphocyte count at the time of the ablation procedure were enrolled in this study (cohort 1). All patients stopped their AADs for a period of at least five half-lives before the catheter ablation. The exclusion criteria were as follows: (1) permanent AF refractory to electrical cardioversion, (2) AF with valvular disease ≥grade 2, and (3) a previous cardiac surgery with a concomitant AF surgery or AFCA.

### Independent AFCA Cohort

For external validation of the association between nutritional status and outcomes of catheter ablation, we used an independent AF ablation cohort that included 805 patients who underwent their first AFCA at Korea University Cardiovascular Center from 2013 to 2016. From the independent data, we enrolled and analyzed 360 patients who had available data on their serum albumin, cholesterol, and total lymphocyte [median age 57 (interquartile range 50-64) years, 20.8% female, and 46.4% paroxysmal AF] for CONUT score evaluation (cohort 2).

### Nutritional Status

A diagnosis of malnutrition was reached using the CONUT score, which is a screening tool for the nutritional status of hospitalized patients (18) and was calculated according to the levels of serum albumin, cholesterol, and total lymphocyte count (Table 1). High CONUT score has been known to have a prognostic impact in patients with chronic cardiac disease including heart failure and AF (6, 14). Also, the score has been associated with outcomes after cardiovascular surgery (19) or interventional procedures including transcatheter aortic valve replacement and percutaneous coronary intervention (20, 21). A score of 0–1 was considered normal (good) nutrition, whereas malnutrition could be classified as mild (2–4) or moderate to severe (≥5).

**Table 1:** Definition of CONUT score.

**Parameters**	**Range and score**			
Serum albumin, g/dl	≥3.50	3.00-3.49	2.50-2.99	<2.50
Score	0	2	4	6
Total Cholesterol, mg/dl	≥180	140-179	100-139	<100
Score	0	1	2	3
Total lymphocyte count, /mm^3^	≥1,600	1,200-1,599	800-1,199	<800
Score	0	1	2	3
Total CONUT score	0-1 points	2-4 points	5-8 points	9-12 points
Degree of malnutrition	Normal	Mild	Moderate	Severe

*CONUT, Controlling nutritional status*.

### Electrophysiological Mapping and Radiofrequency Catheter Ablation

Intracardiac electrograms were recorded using the Prucka CardioLab Electrophysiology system (General Electric Medical Systems, Inc., Milwaukee, WI, USA). Three-dimensional electroanatomic mapping (NavX, St. Jude Medical, Inc., Minnetonka, Minnesota and CARTO, Biosense-Webster, Inc., Diamond Bar, California) was performed using a circumferential pulmonary vein (PV) mapping catheter (Lasso, Biosense-Webster Inc.) through a long sheath (Schwartz left 1, St. Jude Medical, Inc.). Transseptal punctures were performed, and multi-view pulmonary venograms were obtained. The details of the AFCA technique have been described in our previous studies (22, 23). All patients underwent a circumferential PV isolation (CPVI) during the *de novo* procedure. Most patients (87.8%) underwent the creation of cavotricuspid isthmus block during the *de novo* procedure, unless there was atrioventricular conduction disease. As an extra-PV left atrial (LA) ablation, additional linear ablation, including a roof line, posterior inferior line (posterior box lesion), and anterior line, was performed, especially in patients with persistent AF. A left lateral isthmus ablation, right atrial ablation, or complex fractionated electrogram ablation were performed in a minority of patients at the operator's discretion. An open-irrigated tip catheter [Celsius (Johnson & Johnson, Inc., Diamond Bar, CA); NaviStar ThermoCool (Biosense Webster, Inc); ThermoCool SF (Biosense Webster, Inc); ThermoCool SmartTouch (Biosense Webster, Inc); Coolflex (St. Jude Medical, Inc); 30–35 W; 47°C; FlexAbility (St. Jude Medical, Inc); ThermoCool SmartTouch (Biosense Webster, Inc.); and TactiCath (St. Jude Medical, Inc)] was used. Systemic anticoagulation was attained with intravenous heparin to maintain an activated clotting time of 350–400 s during the procedure.

After completion of the protocol-based ablation, the procedure was completed when no recurrence of AF was observed within 10 min after cardioversion with an isoproterenol infusion (5–10 μg/min depending on ß-blocker use, target sinus heart rate of 120 bpm). If further AF triggered or frequent unifocal atrial premature beats were observed due to the effect of isoproterenol, extra-PV foci were ablated using quick 3D activation mapping. We defined an extra-PV LA ablation as an additional linear ablation with or without a complex fractionated electrogram ablation following the CPVI. If recurrent AF or atrial tachycardia (AT) were seen repeatedly under AADs after the *de novo* AFCA, we recommended a repeat AFCA. The detailed technique and strategy for repeat ablation procedures were presented in a previous study (22, 23).

### Post-ablation Management and Follow-Up

We discharged patients not taking AADs except for those who had recurrent extra-PV triggers after the AFCA procedure, symptomatic frequent atrial premature beats, non-sustained atrial tachycardia, or an early recurrence of AF on telemetry during the admission period (28.7%). Electrocardiography was performed for all patients visiting an outpatient clinic 1, 3, 6, and 12 months after AFCA and every 6 months thereafter or whenever symptoms developed. Twenty-four-hour Holter recordings were performed at 3, 6, and 12 months and every 6 months thereafter. Patients who reported episodes of palpitations suggestive of arrhythmia recurrence underwent Holter monitoring or event monitor recordings. AF recurrence was defined as any episode of AF or AT lasting for at least 30 s. Any ECG documentation of AF recurrence within a 3-month blanking period was diagnosed as an early recurrence, and AF recurrence occurring more than 3 months after the procedure was diagnosed as clinical recurrence.

### Statistical Analysis

Continuous variables are summarized as medians (interquartile range) and were compared using the Mann-Whitney *U*-test or Kruskal-Wallis *H*-test. Categorical variables are summarized as frequencies (percentages) and were compared using Fisher's exact test. We used Cochran-Armitage analysis to investigate trends in the complications of AFCA according to CONUT score. Multivariate logistic regression was applied to identify predictors associated with the occurrence of overall and major complications after AFCA. Kaplan–Meier analysis with the log-rank test was used to calculate AF recurrence-free survival over time and to compare recurrence rates across groups. A two-sided *P*-value of < 0.05 was considered statistically significant. The statistical analyses were performed using R version 4.0.2 software (The R Foundation, www.R-project.org).

## Results

### Patient Characteristics and Factors Associated With Malnutrition

Table 2 summarizes the baseline clinical and procedure-related characteristics of the patients undergoing AFCA according to their nutritional status. Of the 3,239 patients included [median age 59 (interquartile range 52-66) years, 26.8% female, and 67.7% paroxysmal AF] (cohort 1), 1,005 (31.0%) had malnutrition; 991 (30.6%) had mild malnutrition (CONUT scores: 2–4), and 14 (0.4%) had moderate-to-severe (CONUT scores: ≥5) malnutrition. Patients with malnutrition were more likely to be older (*P* < 0.001) and male (*P* = 0.014), and had a lower BMI (*P* < 0.001), lower blood pressure (*P* < 0.001), higher CHA_2_DS_2_-VASc score (*P* < 0.001), and more comorbidities than those with normal nutritional status.

**Table 2:** Baseline clinical and procedure-related characteristics of the patients undergoing a *de novo* catheter ablation of atrial fibrillation according to the nutritional status.

**Variables**	**All subjects (*N* = 3,239)**	**Normal nutrition: CONUT 0-1 (*n* = 2,234)**	**Malnutrition: CONUT ≥ 2 (*n* = 1,005)**	***P*-value**
**Clinical characteristics**				
Age, years	59 (52-66)	57 (50-64)	63 (56-69)	<0.001
<65 years	2,249 (69.4)	1,676 (75.0)	573 (57.0)	<0.001
65-74 years	794 (24.5)	461 (20.6)	333 (33.1)	
≥75 years	196 (6.1)	97 (4.3)	99 (9.8)	
Female sex	867 (26.8)	627 (28.1)	240 (23.9)	0.014
Paroxysmal AF	2,184 (67.7)	1,530 (68.9)	654 (65.3)	0.048
BMI, kg/m^2^	24.8 (23.1-26.8)	25.0 (23.3-26.9)	24.6 (22.7-26.6)	<0.001
CONUT score	1 (0-1)	1 (0-1)	2 (2-3)	<0.001
Serum albumin, g/dl	4.3 (4.1-4.5)	4.3 (4.2-4.5)	4.2 (4.0-4.4)	<0.001
Cholesterol, mg/dl	171 (145-198)	186 (164-207)	133 (119-153)	<0.001
Lymphocyte count, /mm^3^	2.03 (1.65-2.47)	2.14 (1.83-2.56)	1.59 (1.30-2.12)	<0.001
eGFR, ml/min/1.73 m^2^	82 (71-120)	83 (72-120)	80 (68-108)	<0.001
Systolic BP, mmHg	120 (110-128)	120 (110-128)	119 (109-128)	0.009
Diastolic BP, mmHg	75 (68-83)	76 (70-83)	73 (66-81)	<0.001
CHA_2_DS_2_-VASc	1 (1-3)	1 (0-2)	2 (1-3)	<0.001
Congestive heart failure	403 (12.4)	247 (11.1)	156 (15.5)	0.001
Hypertension	1509 (46.6)	949 (42.5)	560 (55.7)	<0.001
Diabetes	499 (15.4)	250 (11.2)	249 (24.8)	<0.001
Previous stroke/TIA	375 (11.6)	176 (7.9)	199 (19.8)	<0.001
Previous vascular disease	326 (10.1)	159 (7.1)	167 (16.6)	<0.001
Chronic kidney disease, stage 3–5	306 (9.5)	165 (7.4)	141 (14.0)	<0.001
Anemia	305 (9.4)	130 (5.8)	175 (17.4)	<0.001
LA dimension, mm	41 (37-45)	41 (37-45)	42 (38-46)	<0.001
LVEF, %	64 (59-68)	64 (59-68)	64 (59-69)	0.576
E/Em	9.0 (7.4-12.0)	9.0 (7.1-11.4)	10.0 (8.0-12.7)	<0.001
LA volume index by CT, cm^3^/m^2^	80.5 (66.5-98.1)	79.9 (65.0-95.7)	85.0 (70.0-102./8)	<0.001
Pericardial fat volume, cm^3^	103.1 (71.5-141.5)	102.9 (70.8-140.7)	103.6 (73.1-142.8)	0.209
Mean LA voltage, mV	1.31 (0.82-1.80)	1.31 (0.83-1.82)	1.31 (0.79-1.77)	0.327
Mean LA wall thickness, mm	1.93 (1.73-2.14)	1.93 (1.74-2.14)	1.94 (1.70-2.14)	0.868
**Procedure-related characteristics**				
Procedure time, min	164 (125-200)	165 (126-201)	160 (124-196)	0.062
Fluoroscopy time, min	32 (24-43)	33 (24-44)	32 (24-43)	0.268
Ablation time, sec	4,112 (2,811-5,344)	4,138 (2,829-5,384)	4,083 (2,752-5,270)	0.161
**Ablation lesions**				
CPVI	3,235 (99.9)	2,231 (99.9)	1,004 (99.9)	1.000
CTI	2,819 (87.2)	1,943 (87.2)	876 (87.3)	1.000
POBI	749 (23.2)	525 (23.6)	224 (22.4)	0.464
Anterior line	658 (20.4)	450 (20.2)	208 (20.7)	0.764
Use of contact force sensing catheters	262 (8.1)	179 (8.0)	83 (8.3)	0.867

*Values are presented as median (interquartile range) or n (%)*.

*AF, atrial fibrillation; BP, blood pressure; BMI, body mass index; CT, computed tomography; CONUT, controlling nutritional status; CPVI, circumferential pulmonary vein isolation; CTI, cavo-tricuspid isthmus; E/Em, ratio of the peak mitral flow velocity of the early rapid filling to the early diastolic velocity of the mitral annulus; eGFR, estimated glomerular filtration rate; LA, left atrium; LVEF, left ventricle ejection fraction; POBI, posterior wall box isolation; RA, right atrial; SVC, superior vena cava; TIA, transient ischemic attack*.

### AFCA Complications and Nutritional Status

**Figure 1:**
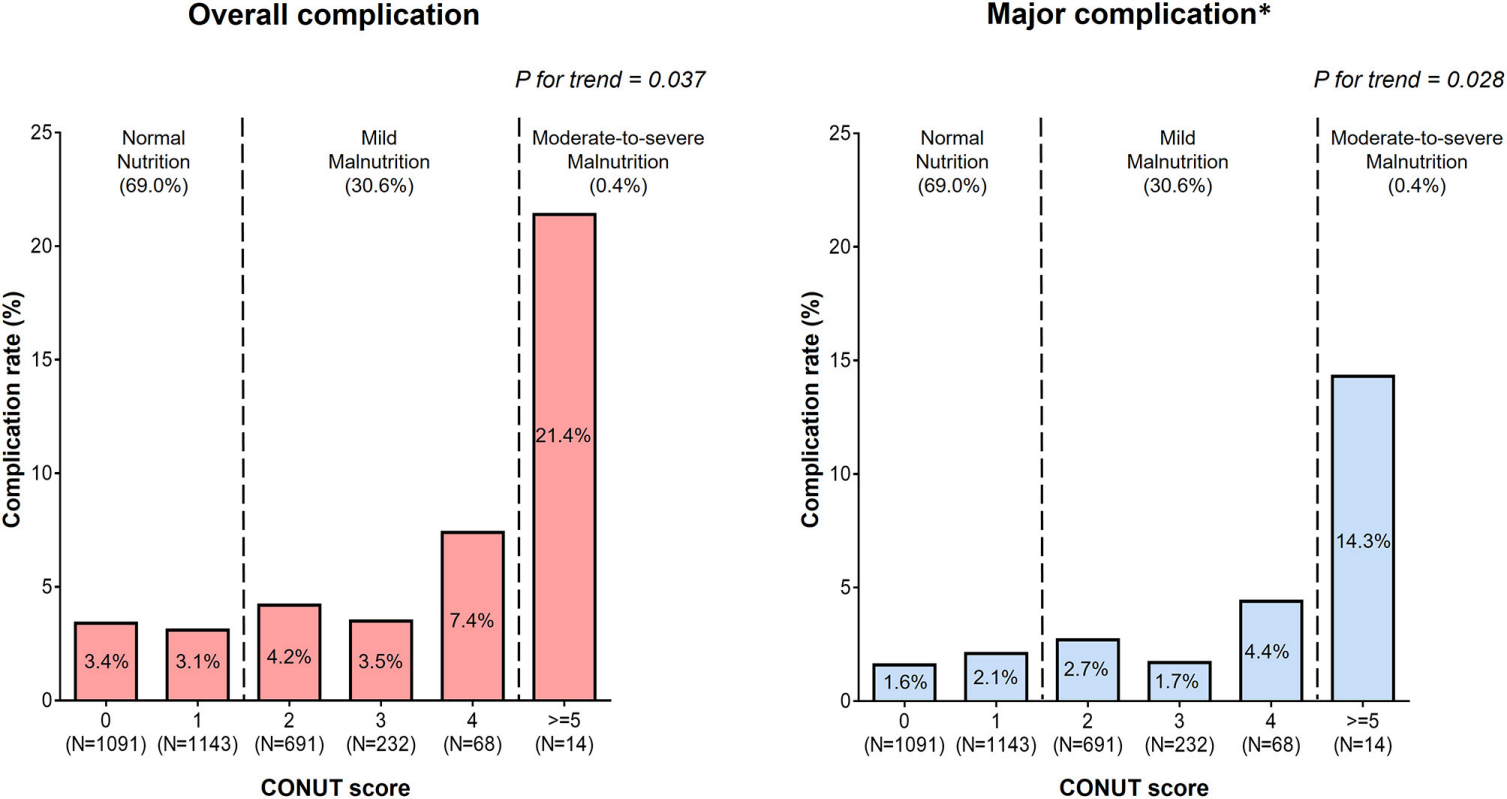
Rates of the overall and major complications according to the CONUT score. ^*^Complications that resulted in permanent injury or death, required intervention, or a prolonged or required hospitalization for more than 48 h.

The rates of complications of AFCA according to CONUT score in cohort 1 are summarized in Figure 1. The overall complication (*P* for trend = 0.037) and major complication rates (*P* for trend = 0.028) were greater in individuals with higher CONUT scores. Detailed information about the complications of AFCA according to nutritional status are presented in Table 3. During the first half of the study period (2009–2015), the overall complication rates after AFCA were 4.0 and 5.1% in those with normal nutrition and malnutrition (CONUT scores: ≥2), respectively. During the second half (2015–2020), the rates slightly decreased to 3.6% for a normal nutrition and 4.9% for a malnutrition. The overall complication rates after AFCA were 3.3% for a normal nutrition, 4.2% for a mild malnutrition, and 21.4% for a moderate-to-severe malnutrition. The major complication rates were 1.9, 2.6, and 14.3% for normal nutrition, mild malnutrition, and moderate-to-severe malnutrition, respectively. In multivariable logistic regression, moderate-to-severe malnutrition was associated with an increased risk of overall (OR 6.456, 95% CI 1.637–25.463, *P* = 0.008) and major complications (OR 5.845, 95% CI 1.164–29.359, *P* = 0.032), compared to normal nutritional status (Table 4). Older age (OR 1.020 per 1-year increase, 95% CI 1.001–1.039), female sex (OR 1.915, 95% CI (1.302–2.817), and higher systolic blood pressure (OR 1.013 per 1-mmHg increase, 95% CI 1.000–1.026) were also independent predictors for the occurrence of complications after AFCA.

**Table 3:** Complications after the de novo catheter ablation of atrial fibrillation.

	**Normal nutrition: CONUT 0-1 (*n* = 2,234)**	**Malnutrition**		
**Complications**		**Overall: CONUT ≥ 2 (*n* = 1,005)**	**Mild: CONUT 2-4 (*n* = 991)**	**Moderate-to-severe: CONUT ≥ 5 (*n* = 14)**
Overall complications	73 (3.3)	45 (4.5)	42 (4.2)	3 (21.4)
Major complication^*^	42 (1.9)	28 (2.8)	26 (2.6)	2 (14.3)
Atrioesophageal fistula	1 (0.0)	2 (0.2)	1 (0.1)	1 (7.1)
Vascular access complication	17 (0.8)	9 (0.9)	8 (0.8)	1 (7.1)
Cardiac tamponade/hemopericardium	28 (1.3)	15 (1.5)	14 (1.4)	1 (7.1)
Pulmonary vein stenosis	4 (0.2)	0 (1.5)	0 (0.0)	0 (0.0)
Phrenic nerve paralysis	3 (0.1)	1 (0.1)	1 (0.1)	0 (0.0)
Stroke/transient ischemic attack	4 (0.2)	2 (0.2)	2 (0.2)	0 (0.0)
Complete atrioventricular block	1 (0.0)	1 (0.1)	1 (0.1)	0 (0.0)
Pericarditis	4 (0.2)	3 (0.3)	3 (0.3)	0 (0.0)
Others^†^	15 (0.7)	12 (1.2)	12 (1.2)	0 (0.0)

*Values are presented as number (%)*.

* *Complications that resulted in permanent injury or death, required intervention for treatment, or a prolonged or required hospitalization for more than 48 h*.

† *Includes pleural effusion, shock due to unknown etiology, sudden cardiac arrest, and sinus node dysfunction*.

**Table 4:** Logistic regression analysis for the predictors of the overall and major complications.

	**Overall complications**				**Major complications** ^*^			
	**Univariable**		**Multivariable**		**Univariable**		**Multivariable**	
**Variables**	**OR (95% CI)**	***P***	**OR (95% CI)**	***P***	**OR (95% CI)**	***P***	**OR (95% CI)**	***P***
**Nutritional status by CONUT**								
Normal nutrition (0-1)	1 (ref)	-	1 (ref)	-	1 (ref)	-	1 (ref)	-
Mild malnutrition (2-4)	1.310 (0.890-1.930)	0.172	1.191 (0.793-1.790)	0.400	1.400 (0.854-2.297)	0.182	1.162 (0.693-1.950)	0.569
Moderate-to-severe malnutrition (≥5)	8.073 (2.205-29.557)	0.002	6.456 (1.637-25.463)	0.008	8.663 (1.880-39.916)	0.006	5.845 (1.164-29.359)	0.032
**Clinical variables**								
Age	1.031 (1.013-1.050)	<0.001	1.020 (1.001-1.039)	0.039	1.039 (1.014-1.063)	0.017	1.030 (1.005-1.056)	0.018
Female sex	2.074 (1.427-3.013)	<0.001	1.915 (1.302-2.817)	0.001	1.746 (1.072-2.843)	0.025	1.518 (0.921-2.502)	0.102
Paroxysmal atrial fibrillation	0.878 (0.596-1.294)	0.512			1.195 (0.708-2.017)	0.506		
BMI (kg/m^2^)	0.947 (0.891-1.008)	0.087	0.963 (0.904-1.026)	0.245	0.925 (0.853-1.003)	0.060	0.951 (0.877-1.032)	0.228
Systolic BP (mmHg)	1.013 (1.000-1.026)	0.051	1.013 (1.000-1.026)	0.043	1.009 (0.993-1.026)	0.285		
Diastolic BP (mmHg)	1.001 (0.985-1.018)	0.893			1.002 (0.981-1.023)	0.866		
Heart failure	0.945 (0.536-1.668)	0.847			1.054 (0.519-2.139)	0.884		
Hypertension	1.327 (0.918-1.918)	0.133			1.291 (0.803-2.074)	0.292		
Diabetes	1.056 (0.64-1.742)	0.831			1.258 (0.683-2.315)	0.461		
Stroke/TIA	1.117 (0.643-1.941)	0.695			0.843 (0.383-1.854)	0.671		
Vascular disease	1.759 (1.062-2.914)	0.028	1.490 (0.869-2.554)	0.147	0.832 (0.358-1.936)	0.670		
Chronic kidney disease, stage 3–5	1.531 (0.981-2.629)	0.123			1.243 (0.589-2.620)	0.568		
Anemia	2.298 (1.423-3.711)	<0.001	1.576 (0.939-2.644)	0.085	2.247 (1.216-4.154)	0.010	1.466 (0.756-2.842)	0.258
**Echocardiographic**								
LA dimension (mm)	0.978 (0.950-1.008)	0.145			0.973 (0.936-1.011)	0.159		
LVEF (%)	1.022 (0.997-1.046)	0.082	1.015 (0.991-1.040)	0.217	1.020 (0.989-1.052)	0.215		
E/Em	1.048 (1.016-1.081)	0.003	1.014 (0.974-1.056)	0.505	1.044 (1.003-1.086)	0.034		
**Computed tomographic**								
Pericardial fat volume (cm^3^)	0.998 (0.994-1.002)	0.378			0.996 (0.991-1.001)	0.132		
Mean LA voltage (mV)	1.205 (0.882-1.647)	0.242			1.180 (0.783-1.777)	0.429		
Mean LA wall thickness (mm)	1.270 (0.707-2.282)	0.424			1.323 (0.622-2.814)	0.468		
**Procedural**								
Procedure time (min)	1.003 (1.000-1.006)	0.079	1.003 (1.000-1.006)	0.060	1.000 (0.995-1.004)	0.832		
Extra-PV LA ablation	1.308 (0.905-1.889)	0.153			1.883 (1.158-3.062)	0.011	1.901 (1.165-3.100)	0.010
Use of contact force sensing catheters	1.175 (0.624-2.216)	0.617			1.481 (0.701-3.127)	0.303		

*Factors significant in the univariable analyses (P <0.10) were entered into the multivariable analyses*.

* *Complications that resulted in permanent injury or death, required intervention for treatment, or a prolonged or required hospitalization for more than 48 hours*.

*BMI, body mass index; CI, confidence interval; E/Em, ratio of the peak mitral flow velocity of the early rapid filling to the early diastolic velocity of the mitral annulus; LA, left atrium; LVEF, left ventricular ejection fraction; TIA, transient ischemic attack; OR, odds ratio*.

**Figure 2:**
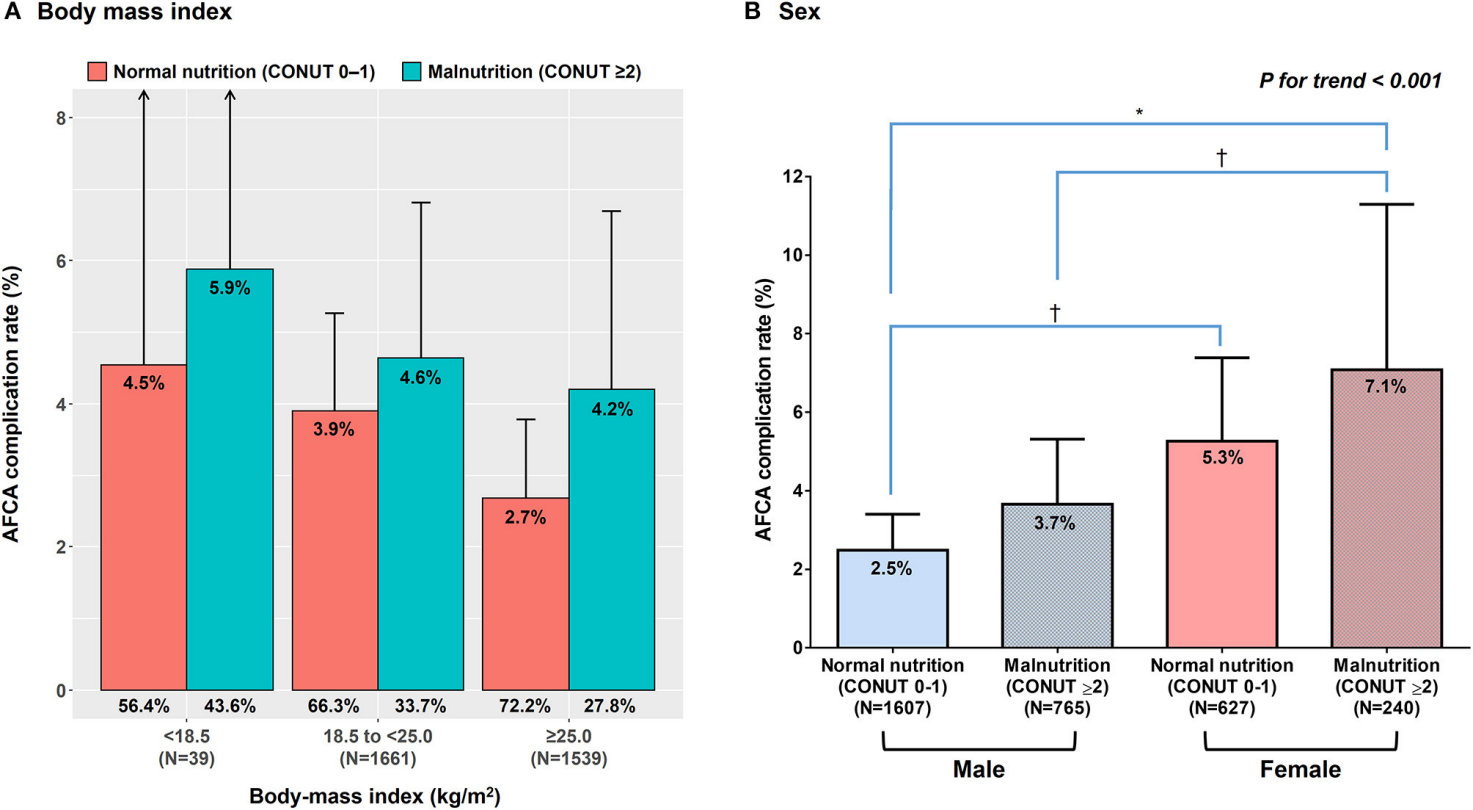
Effects of nutritional status on rates of the overall complications according to body mass index **(A)** and sex **(B)**. *Indicates *P* < 0.001 between the two groups. ^†^Indicates *P* < 0.05 between the two groups. AFCA, atrial fibrillation catheter ablation.

Figure 2 shows the effects of nutritional status on incidences of AFCA complications according to body mass index and sex. Even among overweight or obese patients with body mass indices at least 25 kg/m^2^, malnutrition was prevalent (27.8%). Regardless of body mass index, malnutrition showed trend toward a higher risk of complications compared with normal nutrition (Figure 2A). The rates of overall complications after AFCA were highest (7.1%) in malnourished female patients, followed by 5.3% in normally nourished females, 3.7% in malnourished male patients, and 2.5% in normally nourished males (*P* for trend < 0.001) (Figure 2B).

### External Validation Cohort

Of the 360 patients in the validation cohort (cohort 2), 120 (33.3%) had malnutrition; 117 (32.5%) had mild and 3 (0.8%) had moderate-to-severe malnutrition. 26 (7.2%) had complications after AFCA. The baseline clinical and procedure-related characteristics of the patients in the validation cohort are presented in Supplementary Table 1. The overall complication rates after AFCA were 5.0 and 11.7% for normal nutrition and malnutrition, respectively. Detailed information about the complications of AFCA are presented in Supplementary Table 2. After multivariable adjustment, malnutrition (CONUT ≥ 2) was an independent predictor (OR 2.874, 95% CI 1.174-7.033) for complications after AFCA (Supplementary Table 3). In addition, an increasing CONUT score was associated with a higher risk for complications (OR 1.418 per 1 point increase, 95% CI 1.049-1.916).

### AF Recurrence and Nutritional Status

**Figure 3:**
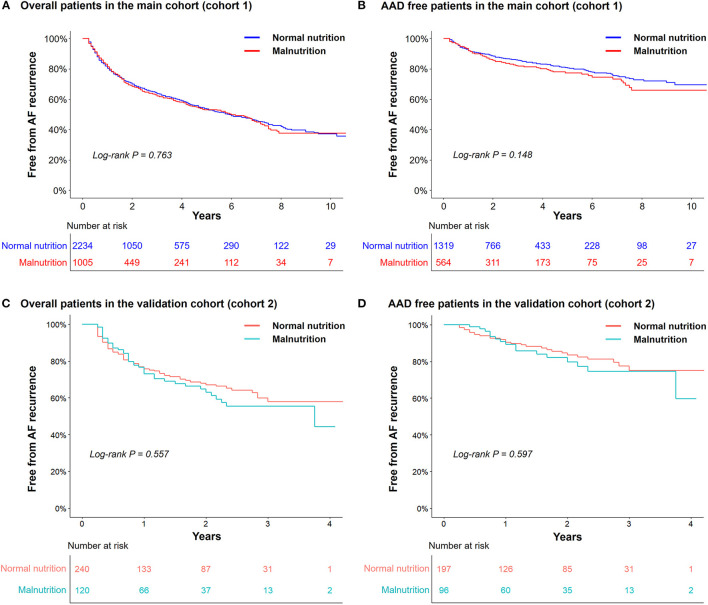
Kaplan–Meier analyses of the atrial fibrillation (AF) recurrence-free survival rate in the cohort 1 (the main cohort) (*n* = 3,239; **A,B**) and in the cohort 2 (the external validation cohort) (*n* = 360, **C,D**). AAD, antiarrhythmic drugs.

Among 3,239 patients in the main cohort (cohort 1), neither the early recurrence rate within 3 months of the AFCA (32.6 vs. 34.4%, *P* = 0.331) nor the clinical recurrence rate (37.0 vs. 36.6%, *P* = 0.829) differed between the normal nutrition and malnutrition groups during the median follow-up of 40 months (interquartile range 18–74 months, Table 5). Kaplan–Meier analysis showed no significant difference in overall AF recurrence (log-rank *P* = 0.763; Figure 3A) or AAD-free AF recurrence (log-rank *P* = 0.148; Figure 3B) between the groups. Repeat ablation procedures were performed in 13.3% of the normally nourished patients and 11.7% of the malnourished patients (*P* = 0.230). In those with a repeat ablation, the proportion of PV reconnections did not differ according to nutritional status (*P* = 0.396, Table 5).

**Table 5:** Clinical rhythm outcomes.

**Outcomes**	**Normal nutrition: CONUT 0-1 (*n* = 2,234)**	**Malnutrition**				
		**CONUT ≥ 2 Overall (*n* = 1,005)**	***P*** ^*^ vs. normal	**Mild: CONUT 2-4 (*n* = 991)**	**Moderate-to-severe: CONUT ≥ 5 (*n* = 14)**	***P*** **^†^ among 0-1, 2-4, and ≥5**
Follow-up duration (months)	41 (19-76)	38 (17-72)	0.070	38 (17-71)	36 (27-79)	0.188
**Post-ABL medication**						
ACEi, or ARB, *n* (%)	697 (31.3)	414 (41.2)	<0.001	407 (41.1)	7 (50.0)	<0.001
Beta blocker, *n* (%)	815 (36.6)	426 (42.4)	0.002	417 (42.1)	9 (64.3)	0.002
Statin, *n* (%)	526 (23.6)	538 (53.5)	<0.001	531 (53.6)	7 (50.0)	<0.001
**AAD use**						
AADs at discharge, *n* (%)	633 (28.3)	295 (29.4)	0.229	291 (29.4)	4 (28.6)	0.633
AADs after 3 months, *n* (%)	811 (36.3)	391 (38.9)	0.414	383 (38.6)	8 (57.1)	<0.001
AADs at final follow-up, *n* (%)	871 (39.8)	422 (42.8)	0.112	415 (42.7)	7 (50.0)	0.229
Early recurrence, *n* (%)	714 (32.6)	340 (34.5)	0.331	332 (34.1)	8 (57.1)	0.115
Recurrence type AT, *n* (% in recur)	212 (29.7)	107 (31.5)	0.484	107 (32.2)	0 (0.0)	0.493
Clinical recurrence, *n* (%)	811 (37.0)	360 (36.5)	0.829	354 (36.4)	6 (42.9)	0.858
Recurrence type AT, *n* (% in recur)	200 (24.6)	117 (32.4)	0.015	113 (31.9)	4 (66.7)	0.017
Cardioversion, *n* (% in recur)	241 (29.7)	97 (26.9)	0.370	95 (26.8)	2 (33.3)	0.590
Repeat AF ablation, *n* (%)	298 (13.3)	118 (11.7)	0.230	116 (11.7)	2 (14.3)	0.435
PV reconnections (% in redo)	208 (69.8)	88 (74.6)	0.396	86 (74.1)	2 (100.0)	0.454
1~2 PV reconnections, *n* (% in redo)	122 (40.9)	44 (37.3)		44 (37.9)	0 (0.0)	
3~4 PV reconnections, *n* (% in redo)	86 (28.9)	44 (37.3)		42 (36.2)	2 (100.0)	

* *Normal nutrition vs. malnutrition Normal nutrition vs. mild malnutrition vs. moderate-to-severe malnutrition*.

† *AAD, antiarrhythmic drug; ABL, ablation; ACEi, angiotensin-converting enzyme inhibitor; AF, atrial fibrillation; ARB, angiotensin receptor blocker; AT, atrial tachycardia; PV, pulmonary vein*.

In the validation cohort of 360 patients (cohort 2), there were consistently no differences in overall AF recurrence or AAD-free AF recurrence between the normal nutrition and malnutrition groups during the median follow-up of 14 months (interquartile range 6-28 months, Figures 3C,D).

## Discussion

### Main Findings

Malnutrition, defined by CONUT score, is relatively common in patients with AF undergoing catheter ablation. In this study, we noted a trend toward a higher risk of AFCA complications in patients with higher CONUT scores. Patients with moderate-to-severe malnutrition (CONUT scores ≥ 5) and females with CONUT scores ≥2 faced a substantially higher risk of complications after AFCA. In an external validation cohort, malnutrition was consistently associated with an increased risk of complications. There was no significant association between nutritional status and AF recurrence after AFCA.

### Malnutrition in Patients With AF

Using the same scoring as that in the present study (CONUT score), Zhu et al. reported that 36.6% of patients undergoing AFCA were malnourished, and 2% had moderate-to-severe malnutrition (15). The results of that study are consistent with those of the present study: 31% of the patients undergoing AFCA had malnutrition, although only 0.4% had moderate-to-severe malnutrition. Several studies have investigated the association of BMI with the clinical outcomes in patients with AF, in which they reported that a BMI of at least 25 kg/m^2^ was associated with a lower risk of a stroke, cardiovascular death, or all-cause mortality, suggesting an “obesity paradox” in AF (5). However, research evaluating the prognostic value of nutritional status beyond BMI is scarce. Raposeiras-Roubín et al. reported that malnutrition, defined using CONUT score, is more prevalent (43.1%) in octogenarian patients with AF and that malnutrition is associated with a higher risk of mortality, strokes, and major bleeding (14). In this study, malnutrition was prevalent even in those with body mass indices at least 25 kg/m^2^. Those findings suggest a discrepancy between BMI and nutritional status and support the importance of assessing and improving nutritional status beyond BMI. The negative impact of malnutrition on the course of AF might be attributable to cardiac cachexia, which leads to the activation of proinflammatory cytokines and neurohormonal dysfunction (24) and a weakened protective effect of adipokines stemming from a reduced fat mass (25).

### Malnutrition and AFCA Outcomes

Numerous studies have investigated relationships between nutritional status and AFCA outcomes using BMI to evaluate nutritional status, and all of them have focused on the efficacy of AFCA, that is, the AF recurrence rate. Baek et al. reported that being overweight and having metabolic syndrome were associated with a higher long-term recurrence rate at 2 years after AFCA in Asian populations in which the proportion of patients with a BMI > 30 kg/m^2^ was only 5.5% (16). Further, Deng et al. reported that being underweight (BMI < 18.5 kg/m^2^) was also associated with a higher AF recurrence (4). However, Bunch et al. reported that there was no significant association between BMI and AF recurrence (26). A recent study in 246 patients undergoing AFCA on the prognostic value of malnutrition using two nutritional status screening tools, including CONUT score, reported that malnutrition was an independent predictor of the recurrence of AF (15). In the present study, we expounded on the prior observations by enrolling a substantially larger number of participants and using longer follow-up durations, showing that the rates of early (<3 months) and clinical (beyond 3 months) AF recurrence did not differ according to nutritional status. However, the complication rates increased significantly as CONUT score increased. Up to 2 out of 10 patients with a CONUT score ≥ 5 sustained complications after AFCA. The trend was consistently observed in overweight or obese patients with AF. Female sex has been reported to be a risk factor for AFCA complications (27), which was consistently shown in this study. The present study showed a substantial risk for AFCA complication in malnourished females. Thus, emphasis should be placed on the assessment of nutritional status in patients with AF who are scheduled to undergo catheter ablation.

### Limitations

This study had several limitations. First of all, the proportion of those with moderate-to-severe malnutrition was too small (0.4%) in this study. Although they were at significantly higher risk of complications in multivariable analyses, this needs to be interpreted carefully and cannot be generalized. This was a retrospective observational cohort study from two centers that included patients using strict selection criteria referred for AF ablation; hence, our findings cannot be used to establish causal relationships. Also, we were not able to fully exclude selection bias. Nutritional status was measured using CONUT score instead of studying body composition or detailed information on diet. Some patients whose CONUT score elements were not available were excluded from this study. We did not compare the prognostic values of other validated screening tools for malnutrition.

## Conclusions

Malnutrition is highly prevalent in patients undergoing AFCA, and patients with malnutrition have a substantially higher risk of procedural complications. Nutritional assessment may provide additional risk stratification for safer AFCA.

## Data Availability Statement

The raw data supporting the conclusions of this article will be made available by the authors, without undue reservation.

## Ethics Statement

The studies involving human participants were reviewed and approved by the institutional review board of the Yonsei University Health System. The patients/participants provided their written informed consent to participate in this study.

## Author Contributions

H-NP and JS contributed to the conception and design of the work and critical revision of the manuscript. DK contributed to the conception and design of the work, interpretation of data, and drafting of the manuscript. JS, YK, HT, T-HK, J-SU, J-IC, and H-NP contributed to the acquisition and analysis of data. BJ, M-HL, and Y-HK contributed to the conception and design of the work. All authors contributed to the article and approved the submitted version.

## Funding

This work was supported by Ministry of Health and Welfare (HI19C0114 and HI21C0011) and by the Basic Science Research Program (NRF-2020R1A2B01001695) run by the National Research Foundation (NRF) of Korea, which is funded by the Ministry of Science, ICT & Future Planning (MSIP).

## Conflict of Interest

The authors declare that the research was conducted in the absence of any commercial or financial relationships that could be construed as a potential conflict of interest.

## Publisher's Note

All claims expressed in this article are solely those of the authors and do not necessarily represent those of their affiliated organizations, or those of the publisher, the editors and the reviewers. Any product that may be evaluated in this article, or claim that may be made by its manufacturer, is not guaranteed or endorsed by the publisher.
