# Supplementary material for: Malnutrition and Risk of Procedural Complications in Patients With Atrial Fibrillation Undergoing Catheter Ablation
Source: Front Cardiovasc Med. 2021 Oct 25;8:736042. doi: 10.3389/fcvm.2021.736042 (PMC8572960; doi:10.3389/fcvm.2021.736042)
Supplement: Supplementary file 1 [file Table_1.docx]

**Supplementary Table 1.** Baseline clinical and procedure-related characteristics of the patients undergoing a *de novo* catheter ablation of atrial fibrillation according to the nutritional status in the external validation cohort (cohort 2) (N=360).

| **Variables** | **All subjects**  **(N=360)** | **Normal nutrition**  **: CONUT 0-1**  **(n=240)** | **Malnutrition**  **: CONUT ≥2**  **(n=120)** | **P value** |
| --- | --- | --- | --- | --- |
| **Clinical characteristics** |  |  |  |  |
| Age, years | 57 (50-64) | 56 (49-62) | 60 (53-67) | <0.001 |
| <65 years | 278 (77.2) | 196 (81.7) | 82 (68.3) | 0.014 |
| 65-74 years | 63 (17.5) | 35 (14.6) | 28 (23.3) |  |
| ≥75 years | 19 (5.3) | 9 (3.8) | 10 (8.3) |  |
| Female sex | 76 (21.1) | 55 (22.9) | 21 (17.5) | 0.294 |
| Paroxysmal AF | 167 (46.4) | 116 (48.3) | 51 (42.5) | 0.350 |
| BMI, kg/m^2^ | 24.8 (22.8-26.7) | 24.9 (23.0-26.9) | 24.2 (22.7-26.2) | 0.087 |
| CONUT score | 1 (0-2) | 0 (0-1) | 2 (2-3) | <0.001 |
| Serum albumin, g/dL | 4.4 (4.2-4.6) | 4.5 (4.3-4.6) | 4.4 (4.1-4.6) | 0.009 |
| Cholesterol, mg/dL | 179 (154-202) | 189 (171-209) | 142 (124-169) | <0.001 |
| Lymphocyte count, /mm^3^ | 1.92 (1.48-2.35) | 2.07 (1.77-2.53) | 1.41 (1.07-1.64) | <0.001 |
| CHA_2_DS_2_-VASc | 1 (1 – 3) | 1 (0 – 2) | 2 (1 – 3) | <0.001 |
| Congestive heart failure | 7 (1.9) | 3 (1.2) | 4 (3.3) | 0.345 |
| Hypertension | 125 (34.7) | 73 (30.4) | 52 (43.3) | 0.021 |
| Diabetes | 33 (9.2) | 11 (4.6) | 22 (18.3) | <0.001 |
| Previous stroke/TIA | 26 (7.2) | 13 (5.4) | 13 (10.8) | 0.098 |
| Previous vascular disease | 26 (7.2) | 11 (4.6) | 15 (12.5) | 0.012 |
| LA dimension, mm | 42 (38-45) | 42 (38-45) | 42 (38-45) | 0.381 |
| LVEF, % | 57 (55-58) | 57 (52-57) | 57 (54-57) | 0.735 |
| E/Em | 8.6 (6.7-10.7) | 8.1 (6.4-10.0) | 9.5 (7.3-12.0) | <0.001 |
| **Procedure-related characteristics** |  |  |  |  |
| CPVI | 360 (100.0) | 240 (100.0) | 120 (100.0) | 1.000 |
| CFAE-guided ablation | 159 (44.2) | 105 (43.8) | 54 (45.0) | 0.910 |
| Roof line ablation | 63 (17.5) | 39 (16.2) | 24 (20.0) | 0.462 |
| Anterior line ablation | 48 (13.3) | 35 (14.6) | 13 (10.8) | 0.411 |
| Posteroinferior line ablation | 11 (3.1) | 8 (3.3) | 3 (2.5) | 0.914 |
| Perimitral isthmus line ablation | 29 (8.1) | 15 (6.2) | 14 (11.7) | 0.115 |
| CTI ablation | 243 (67.5) | 161 (67.1) | 82 (68.3) | 0.905 |
| SVC to RA septum | 25 (6.9) | 13 (5.4) | 12 (10.0) | 0.164 |

Values are presented as median (interquartile range) or n (%).

AF, atrial fibrillation; BMI, body mass index; CFAE, complex fractionated atrial electrogram; CONUT, controlling nutritional status; CPVI, circumferential pulmonary vein isolation; CTI, cavo-tricuspid isthmus; E/Em, ratio of the peak mitral flow velocity of the early rapid filling to the early diastolic velocity of the mitral annulus; LA, left atrium; LVEF, left ventricle ejection fraction; RA, right atrial; SVC, superior vena cava; TIA, transient ischemic attack.
