# Supplementary material for: Malnutrition and Risk of Procedural Complications in Patients With Atrial Fibrillation Undergoing Catheter Ablation
Source: Front Cardiovasc Med. 2021 Oct 25;8:736042. doi: 10.3389/fcvm.2021.736042 (PMC8572960; doi:10.3389/fcvm.2021.736042)
Supplement: Supplementary file 2 [file Table_2.docx]

**Supplementary Table 2.** Complications after the de novo catheter ablation of atrial fibrillation in the external validation cohort (cohort 2) (N=360).

|  | **Normal nutrition**  **: CONUT 0-1**  **(n=240)** | **Malnutrition** | | |
| --- | --- | --- | --- | --- |
| **Complications** |  | **Overall**  **: CONUT ≥2**  **(n=120)** | **Mild**  **: CONUT 2-4**  **(n=117)** | **Moderate-to-severe**  **: CONUT ≥5**  **(n=3)** |
| Overall complications | 12 (5.0) | 14 (11.7) | 13 (11.1) | 1 (33.3) |
| Major complication* | 8 (3.3) | 12 (10.0) | 11 (9.4) | 1 (33.3) |
| Atrioesophageal fistula | 0 (0.0) | 0 (0.0) | 0 (0.0) | 0 (0.0) |
| Vascular access complication | 2 (0.8) | 3 (2.5) | 3 (2.6) | 0 (0.0) |
| Cardiac tamponade/hemopericardium | 3 (1.3) | 6 (5.0) | 5 (0.0) | 1 (33.3) |
| Pulmonary vein stenosis | 1 (0.4) | 0 (0.0) | 0 (0.0) | 0 (0.0) |
| Phrenic nerve paralysis | 3 (1.3) | 3 (2.5) | 3 (0.0) | 0 (0.0) |
| Stroke/transient ischemic attack | 1 (0.4) | 0 (0.0) | 0 (0.0) | 0 (0.0) |
| Complete atrioventricular block | 1 (0.4) | 0 (0.0) | 0 (0.0) | 0 (0.0) |
| Pericarditis | 0 (0.0) | 1 (0.8) | 1 (0.0) | 0 (0.0) |
| Others† | 4 (1.7) | 4 (3.3) | 4 (0.0) | 0 (0.0) |

Values are presented as number (%).

*Complications that resulted in permanent injury or death, required intervention for treatment, or a prolonged or required hospitalization for more than 48 hours.

†Includes pulmonary edema, urinary bleeding, and sinus node dysfunction.
