# Supplementary material for: Malnutrition and Risk of Procedural Complications in Patients With Atrial Fibrillation Undergoing Catheter Ablation
Source: Front Cardiovasc Med. 2021 Oct 25;8:736042. doi: 10.3389/fcvm.2021.736042 (PMC8572960; doi:10.3389/fcvm.2021.736042)
Supplement: Supplementary file 3 [file Table_3.docx]

**Supplementary Table 3.** Logistic regression analysis for the predictors of the overall complications in the external validation cohort (cohort 2) (N=360).

|  | **Univariable** | | **Multivariable model 1*** | | **Multivariable model 2**† | |
| --- | --- | --- | --- | --- | --- | --- |
| **Variables** | **OR (95% CI)** | **P** | **OR (95% CI)** | **P** | **OR (95% CI)** | **P** |
| **Malnutrition (CONUT ≥2)** | 2.509 (1.122-5.611) | 0.025 | 2.874 (1.174-7.033) | 0.021 |  |  |
| **CONUT** s**core (per 1 increase)** | 1.385 (1.057-1.814) | 0.018 |  |  | 1.418 (1.049-1.916) | 0.023 |
| **Clinical variables** |  |  |  |  |  |  |
| Age (year) | 1.028 (0.989-1.069) | 0.157 | 0.997 (0.95-1.045) | 0.910 | 0.997 (0.952-1.045) | 0.907 |
| Female sex | 2.110 (0.901-4.92) | 0.086 | 2.595 (1.075-6.330) | 0.036 | 2.373 (0.923-6.101) | 0.073 |
| Paroxysmal atrial fibrillation | 1.633 (0.72-3.660) | 0.234 | 1.344 (0.498-3.622) | 0.560 | 1.377 (0.505-3.752) | 0.532 |
| BMI (kg/m^2^) | 1.015 (0.896-1.151) | 0.809 | 0.984 (0.850-1.139) | 0.829 | 0.996 (0.858-1.155) | 0.953 |
| Heart failure | - | 0.987 | - |  | - |  |
| Hypertension | 1.676 (0.750-3.745) | 0.208 | 1.168 (0.448-3.042) | 0.751 | 1.147 (0.439-2.998) | 0.780 |
| Diabetes | 1.322 (0.375-4.661) | 0.664 | 0.826 (0.212-3.226) | 0.783 | 0.979 (0.259-3.710) | 0.976 |
| Stroke/TIA | - | 0.990 | - |  | - |  |
| Vascular disease | - | 0.990 | - |  | - |  |
| **Echocardiographic** |  |  |  |  |  |  |
| LA dimension (mm) | 1.088 (1.018-1.164) | 0.013 | 1.086 (0.998-1.194) | 0.088 | 1.094 (1.015-1.178) | 0.018 |
| LVEF (%) | 0.989 (0.932-1.050) | 0.724 | 1.009 (0.947-1.076) | 0.778 | 1.011 (0.948-1.078) | 0.748 |
| E/Em | 1.069 (0.984-1.162) | 0.114 | 0.998 (0.889-1.120) | 0.971 | 0.995 (0.888-1.116) | 0.936 |

*The CONUT score (≥2 or 0-1) was included as a binary variable in the model. †The CONUT score was included as a continuous variable in the model.

BMI, body mass index; CI, confidence interval; E/Em, ratio of the peak mitral flow velocity of the early rapid filling to the early diastolic velocity of the mitral annulus; LA, left atrium; LVEF, left ventricular ejection fraction; TIA, transient ischemic attack; OR, odds ratio.
